# Editorial: Current trends and future perspectives about liquid biopsy

**DOI:** 10.3389/fgene.2023.1345876

**Published:** 2023-12-14

**Authors:** Elisa Frullanti, María José Serrano

**Affiliations:** ^1^ Cancer Genomics and Systems Biology Laboratory, Department of Medical Biotechnologies, University of Siena, Siena, Italy; ^2^ Department of Medical Biotechnologies, Med Biotech Hub and Competence Centre, University of Siena, Siena, Italy; ^3^ GENYO Centre for Genomics and Oncological Research, Pfizer/University of Granada/Andalusian Regional Government, Liquid Biopsy and Cancer Interception Group, PTS Granada, Granada, Spain; ^4^ Biomedical Research Institute IBS-Granada, Granada, Spain; ^5^ Integral Oncology Division, Virgen de las Nieves University Hospital, Granada, Spain

**Keywords:** human cancer, liquid biopsy, cancer biomarkers, circulating cancer molecules, disease monitoring

## Introduction

Despite rapid advancements in cancer screening and therapies, many cancer patients still succumb to the disease. The molecular characteristics of tumors are commonly assessed using surgical specimens or biopsy samples. However, biopsies only sample a portion of the tumor and might not fully represent its heterogeneity, providing incomplete information about the genetic variability of a patient’s cancer. Additionally, it is impractical for patients to undergo multiple biopsies of primary and metastatic lesions as the tumor progresses.

During the last years, to answer the need of a more accessible approach for tumor genetic analysis, liquid biopsy has emerged as an innovative, minimally-invasive and efficient opportunity of detecting and monitoring cancer in several body fluids instead of tumor tissue. Furthermore, thanks to this approach, we are able to take more blood samples over time, informing about the type of molecular changes going on in a tumor. Circulating tumor DNA (ctDNA), Circulating tumor cells (CTCs), RNA (mRNA and microRNA), microvesicles, including exosomes and tumor educated platelets represent a source of genomic information in cancer patients reflecting all subclones present in primary and metastatic lesions allowing sequential monitoring of disease evolution.

This Research Topic aimed to update the currently available information concerning liquid biopsy, the key features and their applications in oncology highlighting the technological challenges and the hurdles we need to overcome to finally see the next era of cancer care.

## Most innovative features of liquid biopsy

The reviews and original articles published in the present Research Topic updated about the key and most innovative features of liquid biopsy and their applications in oncology. In particular, Liu et al. assessed the potential utility of miRNAs as biomarkers and highlighted certain promising candidates for liquid biopsy approach in the diagnosis and management of breast cancer that may optimize the patient outcome. They highlighted how some miRNAs, including miR-21 and miR-155, were found to play an important role in breast cancer progression as well as in breast cancer management.

Similarly, Palmieri et al. assessed the diagnostic performance of cell free DNA analysis for the detection of KRAS mutations in non-small cell lung cancer, compared to tissues, through a meta-analysis and systematic review. The analysis of 40 studies including more than 2,800 NSCLC patients revealed that the detection of KRAS mutation in cfDNA has an adequate diagnostic accuracy and might be a valid alternative for molecular analysis when tumor biopsy or cytological specimens are not available.

Di Sario et al. highlighted the importance of an integrated multi-omic, multi-analyte approach of liquid biopsy in the research of novel prognostic and predictive biomarkers for cancer as well as in the monitoring of the course of the disease.

Finally, Zhao et al. focused their attention on ctDNA analysis for prognosis prediction in Chinese newly diagnosed follicular lymphoma patients with interesting results. The most commonly mutated genes were CREBBP, KMT2D, STAT6, CARD11, PCLO, EP300, BCL2, and TNFAIP3. Patients with detectable ctDNA mutation tended to present with advanced stages. In particular, Progression-Free Survival resulted shorter in patients with KMT2D, EP300 and STAT6 mutations.

Beyond nucleic acids, the presence of Circulating Tumor Cells (CTCs) has been acknowledged as an independent prognostic marker in various solid tumors, including breast, colon, and prostate cancer. The prognostic value was demonstrated 20 years ago by Cristofanilli et al. (2004) and Gaforio et al. (2003) in breast cancer patients (Gaforio et al., 2003; Cristofanilli et al., 2004). Recent approaches have aimed to comprehend the biology of these CTCs. Consequently, the assessment of circulating tumor cells (CTCs) allows for repeated sampling to identify the genomic instability of the tumor. Identifying EGFR and KRAS mutations is crucial in guiding the treatment of non-small cell lung cancer (NSCLC) patients undergoing EGFR tyrosine kinase inhibitors and colorectal cancer patients receiving anti-EGFR therapy, respectively. The comparison of mutations between CTCs and corresponding primary or metastatic tumor tissue has generated significant interest.

Meanwhile, Extracellular Vesicles (EVs) circulating tumor-derived endothelial cells (CTECs), and tumor-educated blood platelets (TEPs), considered as carriers of molecular information shed by tumors, have gained attention due to their potential in providing valuable genetic and proteomic data for cancer diagnostics and monitoring (Mehran et al. 2014; Liu et al., 2020; Yu et al., 2021) Including examples of these different LBs, widely present in plasma, urine, ascites, and other body fluids, broadens our understanding of liquid biopsy technologies and their applications in diverse cancer types.

## Conclusion

In conclusion, this Research Topic highlights the importance of liquid biopsy approach in revolutionizing cancer precision medicine. The key findings discussed herein demonstrated the increasing significance of the multi-omics and multi-markers analysis for the identification of new biomarkers, which may prove useful for diagnosis, prognosis and management of cancer patients.
